# The Hong Kong Society of Rheumatology consensus recommendations for the management of gout

**DOI:** 10.1007/s10067-023-06578-9

**Published:** 2023-04-04

**Authors:** Ronald ML Yip, Tommy T Cheung, Ho So, Julia PS Chan, Carmen TK Ho, Helen HL Tsang, Carrel KL Yu, Priscilla CH Wong, for the Hong Kong Society of Rheumatology

**Affiliations:** 1 Tung Wah Group of Hospitals Integrated Diagnostic and Medical Centre, Kwong Wah Hospital, 25, Waterloo Road, Kowloon, Hong Kong; 2 Rheumatology Centre, Department of Medicine, Hong Kong Sanatorium & Hospital, Happy Valley, Hong Kong; 3 Department of Medicine and Therapeutics, The Chinese University of Hong Kong, Ma Liu Shui, Hong Kong; 4 Division of Rheumatology and Clinical Immunology, Queen Mary Hospital, Pok Fu Lam, Hong Kong; 5 Hong Kong Autoimmune and Rheumatic Diseases Centre, Central, Hong Kong

**Keywords:** Consensus, Gout, Hong Kong Society of Rheumatology, Hyperuricemia, Recommendations

## Abstract

Gout is one of the most common noncommunicable diseases in Hong Kong. Although effective treatment options are readily available, the management of gout in Hong Kong remains suboptimal. Like other countries, the treatment goal in Hong Kong usually focuses on relieving symptoms of gout but not treating the serum urate level to target. As a result, patients with gout continue to suffer from the debilitating arthritis, as well as the renal, metabolic, and cardiovascular complications associated with gout. The Hong Kong Society of Rheumatology spearheaded the development of these consensus recommendations through a Delphi exercise that involved rheumatologists, primary care physicians, and other specialists in Hong Kong. Recommendations on acute gout management, gout prophylaxis, treatment of hyperuricemia and its precautions, co-administration of non-gout medications with urate-lowering therapy, and lifestyle advice have been included. This paper serves as a reference guide to all healthcare providers who see patients who are at risk and are known to have this chronic but treatable condition.

## Introduction

The global incidence and prevalence of gout has substantially increased over the past decade [1], and this is no exception in Hong Kong. A local population-based study reported an increase in the crude incidence rate of gout from 113.05/100,000 person-years in 2006 to 211.62/100,000 person-years in 2016 [2]. Similarly, the crude prevalence of gout has risen from 1.56% in 2006 to 2.92% in 2016 [2]. Population aging has contributed significantly to the increased incidence and prevalence of gout in Hong Kong. Over the past 20 years, the number of Hong Kong people aged 65 years or above increased from 12.5% to 16.1%. In addition, modifiable risk factors for gout and hyperuricemia, such as regular alcohol consumption, greater meat intake, and obesity, have been increasing. As a result, the Hong Kong government has included gout, alcohol drinking, and obesity among the target non-communicable diseases in its campaign programs for primary prevention [3]. However, the management of gout in Hong Kong is yet to be improved.

The treatment goal usually focuses on relieving symptoms of arthritis but not treating the underlying hyperuricemia. Despite the availability and demonstrated efficacy of urate-lowering therapy (ULT) in reducing serum uric acid (SUA) levels, only 25.55% of all gout patients received ULT and only 35.8% of these patients achieved the target SUA level of < 0.36 mmol/L (6 mg/dL). Almost all gout patients received allopurinol because it is subsidized by the government. Other unsubsidized ULT options, including febuxostat, were prescribed to < 1% of all gout patients [2]. Resolving the unmet need for the management of hyperuricemia and gout is challenging, because this includes certain patient factors, such as pharmacologic compliance, adverse reactions, and lack of health literacy, and healthcare provider factors, such as under-recognition of over-producers and/or under-excretors, as well as knowledge gaps in gout management [4, 5].

Although several international guidelines for the management of gout are available, there are certain differences according to their ethnic, genetic, social, and cultural background, clinical practice, and the availability of medicine [6–10]. The Hong Kong Society of Rheumatology (HKSR), a professional organization that has been at the helm of training and improving the care of patients with rheumatic diseases in Hong Kong, spearheaded the development of these consensus recommendations aiming at improving the acute and long-term management of gout, particularly focusing on aspects related to the clinical practice in Hong Kong. It is hoped that these recommendations can offer guidance to not only primary care practitioners, but also specialists who regularly see patients who are at risk for gout and its complications.

## Method

### Steering committee

Eight core members of the Gout Special Interest Group (Gout SIG) from the HKSR met through several rounds of teleconferences from July 2020 onwards to discuss proposed consensus recommendations relevant to the management of gout in Hong Kong. Priority areas covered during the meetings included: (1) acute gout management, (2) gout prophylaxis, (3) treatment of hyperuricemia, (4) co-administration of non-gout medications with ULT, and (5) lifestyle modifications aimed at reducing SUA. A total of four overarching principles and 24 major recommendations were reviewed and tweaked. A systematic literature search was concurrently performed to identify and grade the relevant level of evidence for each recommendation.

### Literature search and grading of evidence

Evidence for the consensus statements was identified through an online search of the Medline database with the search terms “acute gout therapy,” “clinical practice guidelines,” “gout,” “gout prophylaxis,” “hyperuricemia,” “lifestyle modification,” “precaution,” “urate lowering therapy,” and “non-pharmacological management” and other relevant key words in the statements. Studies included were published in the past 10 years including systematic reviews, meta-analyses, clinical trials, cohort studies, and clinical practice guidelines published in English. The level of evidence of each statement was evaluated based on the Oxford Centre for Evidence-Based Medicine system (Table 1) [11].

**Table 1:** The Oxford Centre for Evidence-Based Medicine system for grading level of evidence [11]

Level	Type of evidence
1A	Systematic review (with homogeneity) of RCTs
1B	Individual RCT (with narrow confidence intervals)
1C	All or none study
2A	Systematic review (with homogeneity) of cohort studies
2B	Individual cohort study (including low-quality RCT, e.g., < 80% follow-up)
2C	“Outcomes” research; ecological studies
3A	Systematic review (with homogeneity) of case–control studies
3B	Individual case–control study
4	Case series (and poor-quality cohort and case–control study)
5	Expert opinion without explicit critical appraisal or based on physiology bench research or “first principles”

*RCT* Randomized controlled trial

### Consensus formation

Practicing rheumatologists registered as full members of the HKSR and a selected group of family physicians, renal physicians, and internists with special interest and extensive clinical experience in gout management were invited to participate in a modified Delphi exercise. Each overarching statement and all major statements were sent to the participants for voting through anonymized online surveys. The eight core members of the steering committee were excluded from the voting sessions to avoid bias and skewing of the results. The participants were provided with the results of the literature search, explanatory notes, and the level of evidence grading. For each statement, the members were asked to share their level of agreement (strongly agree, agree, neutral, disagree, and strongly disagree) using an online platform hosted by MIMS, an independent medical communications company. They were also invited to give feedback on the statements, indicate reasons for disagreement, and suggest edits or improvements. Consensus was defined as an agreement (strongly agree or agree) of ≥ 80% by the Delphi participants. The anonymized voting results and feedback were then communicated to the core members. Statements that did not meet consensus were then either removed or modified and redeployed to the Delphi participants along with brief elucidatory notes for the subsequent rounds of voting until consensus was reached.

## Results

A total of 84 full HKSR members and 11 practitioners from various specialties were invited. Overall, 60 participated in the Delphi process. The group consisted of 83% rheumatologists, 9% renal medicine specialists, 5% general practitioners, and 3% internists. Among the HKSR members, the response rate was 60% (50 of 84 eligible practicing rheumatologists). After two rounds of voting, consensus was reached for all overarching statements and for 20 of the 24 major statements. The results of the voting for rounds 1 and 2 are summarized in Tables 2 and 3, respectively.

**Table 2:** First round voting results of the major consensus statements on gout management

Statements	Level of evidence	Agreement (%)
Overarching principles		
• “Where possible, every patient with gout should be educated about the pathophysiology of the disease, treatment options for gouty arthritis, its associated comorbidities, and indications of urate-lowering agents.”	NA	97
• “Every patient with gout should receive advice from their healthcare professionals regarding lifestyle modification and its role in the management of gout.”	NA	100
• “Apart from rheumatologists, non-rheumatologist physicians and/or general practitioners should be responsible for managing patients with gout. However, rheumatologists should provide specialist care for patients with gouty arthritis refractory to treatment with first-line therapies or those who fail to achieve the treatment target despite appropriate use of ULT.”	NA	95
• “Every patient with gout should be systematically screened for associated comorbidities and cardiovascular risk factors.”	NA	97
Acute gout management		
• “Acute flares of gout should be treated as early as possible, preferably within 12 h. Patients should be educated to self-medicate at the first warning symptoms.”	1B	95
• “Colchicine, NSAIDs, or glucocorticoids (oral, intra-articular, or intramuscular) are recommended as first-line therapy for gout flares.”	1B	88
• “The choice of drug(s) should be based on the presence of comorbidities, patient's previous response to treatments, the severity of flares, and the number and type of joint(s) involved.”	1B	98
• “Use topical ice as an adjuvant therapy for pain relief.”	1B	80
• “IL-1 inhibitors can be considered for treatment of gout flare in patients who have inadequate response to or are contraindicated for standard treatment (including colchicine, NSAIDs, and/or glucocorticoids). IL-1 inhibitors are contraindicated in patients with active infection.”	1B	82
Gout prophylaxis		
• “Prophylaxis with colchicine 0.5 mg once or twice daily for 3–6 months is recommended during initiation or up-titration of ULT.”	2B	90
• “In patients who cannot tolerate colchicine, a low-dose NSAID can be used provided there are no contraindications.”	2B	75
• “IL-1 inhibitors may be considered as second-line treatment for gout prophylaxis in patients who are contraindicated for colchicine or NSAIDs. However, their costs and putative infection risks may preclude their use as first-line prophylactic agent.”	2B	68
Indications for ULT		
• “ULT should be discussed with all patients diagnosed with gout.”	5	87
• “ULT should not be routinely recommended on the first attack of gout in patients without comorbidities.”	5	87
• “ULT should be recommended to all gout patients with tophi, radiographic damage related to gout, OR recurrent attacks (≥ 2 times per year).”	1B	100
• “ULT can be considered in patients with gout after the first attack with urolithiasis, more than one flare but with infrequent attacks per year, or in those with renal impairment.”	1B	97
• “ULT may be considered in patients with gout after the first attack with very high serum urate level (> 0.54 mmol/L [9 mg/dL]) or young onset age (age < 40 years).”	1B	92
Treatment target of ULT		
• “For patients on ULT, serum urate level should be monitored and maintained below 0.36 mmol/L (6 mg/dL). A target SUA of < 0.30 mmol/L (5 mg/dL) is recommended for patients with tophaceous gout.”	3	95
• “When the clinical tophi have resolved, the serum urate level may be maintained between 0.30 mmol/L (5 mg/dL) and 0.36 mmol/L (6 mg/dL).”	3	87
Precautions in prescribing allopurinol		
• “Allopurinol should be avoided in patients who tested positive for HLA‑B*5801 allele.”	2B	97
• “Screening for the HLA-B*5801 allele should be considered for some patients of Asian descent (e.g., Han Chinese, Korean, Thai) and for African American patients, and patients with risk factors to develop allopurinol-induced SCAR, before starting allopurinol. Risk factors included patients who are ≥ 60 years old or with renal insufficiency (CKD stage ≥ 3).”	2B	92
• “Allopurinol desensitization may be considered for patients with a prior mild allergic response to allopurinol who cannot be treated with other ULT and negative HLA-B*5801 test.”	5	72
Use of ULT		
• “A xanthine oxidase inhibitor (allopurinol or febuxostat) is the preferred agent for all patients with gout.”	1A	100
• “If the targeted serum urate level cannot be achieved by allopurinol, febuxostat should be considered. Alternatively, combination therapy with a uricosuric agent can be considered in patients without severe renal impairment (GFR < 30 mL/min).”	1A	93
Co-administration of non-gout medications with ULT		
• “Calcium channel blockers and losartan are preferred over other antihypertensive drugs e.g., loop or thiazide diuretics, beta-blocker, angiotensin-converting enzyme inhibitors, and non-losartan angiotensin II receptor blockers in patients with gout.”	3	77
• “For patients with gout who are receiving low-dose aspirin (< 300 mg daily) alone or in combination with clopidogrel or ticagrelor for prevention or treatment of cardiovascular disease, serum uric acid levels should be regularly monitored and appropriate ULT adjustment should be made accordingly.”	5	67
Lifestyle modification		
• “Patients should be advised to avoid alcohol, fructose, or sugar-sweetened beverages, and decrease dietary protein source from meat and seafood.”	2B	88
• “Weight reduction is recommended if BMI > 25 kg/m^2^.”	2B	97

*BMI* Body mass index; *CKD* Chronic kidney disease; *GFR* Glomerular filtration rate; *HLA* Human leukocyte antigen; *IL-1* Interleukin-1; *NSAID* Non-steroidal anti-inflammatory drug; *SCAR* Severe cutaneous adverse reaction; *SUA* Serum uric acid; *ULT* Urate-lowering therapy

**Table 3:** Second round voting results of the major consensus statements on gout management

Statements	Level of evidence	Agreement
Gout prophylaxis		
• “In patients who cannot tolerate colchicine, a low-dose NSAID can be considered as an alternative for prophylaxis after weighing the risks and benefits.”	2B	84

*NSAID* Non-steroidal anti-inflammatory drug

### Overarching principles

These unifying themes include general recommendations on lifestyle and educational interventions, and the adequate management of comorbidities. These endeavors are typically included in health literacy campaigns to prevent and control the symptoms and complications of gout [3].

A. *“Where possible, every patient with gout should be educated about the pathophysiology of the disease, treatment options for gouty arthritis, its associated comorbidities, and indications of urate-lowering agents.”*

Most studies cite suboptimal understanding of gout and its treatment as a common barrier to gout care. Physicians should effectively communicate to their patients the direct causal role of hyperuricemia to gout and gouty arthritis and its comorbidities, as well as the treatable nature of the disease [12].

B. *“Every patient with gout should receive advice from their healthcare professionals regarding lifestyle modification and its role in the management of gout.”*

Although gout is considered as a chronic and potentially progressive condition requiring long-term management, many people with gout falsely regard gout as an episodic illness and are thus prone to poor medication compliance and lack of treatment response or paradoxical attacks [12]. It is thus crucial to discuss the treatment plan with patients to lessen the risk of gout attacks and the metabolic complications associated with hyperuricemia [3].

III. *“Apart from rheumatologists, non-rheumatologist physicians and/or general practitioners should be responsible for managing patients with gout. However, rheumatologists should provide specialist care for patients with gouty arthritis refractory to treatment with first-line therapies or those who fail to achieve the treatment target despite appropriate use of ULT.”*

The continued partnership between patients and primary care and other health professionals plays an important role in monitoring and improving the management of gout in Hong Kong [3]. Rheumatologists are often called in to reinforce the management of gout patients; they determine whether gout is the cause of arthritis and provide specialist advice to further enhance education on adherence and continued monitoring of the signs and symptoms of gout.

IV. *“Every patient with gout should be systematically screened for associated comorbidities and cardiovascular risk factors.”*

A systematic review by van Durme et al. revealed that hyperuricemia appears to be a risk indicator and is a component of the metabolic syndrome, rather than an independent risk factor for cardiovascular disease, but may nonetheless contribute to a slight increase in cardiovascular risk. Renal disease, on the other hand, appears to be markedly prevalent among hyperuricemic patients and may therefore need to be considered when administering ULT to at-risk patients [13].

### Acute gout management

**Fig. 1:**
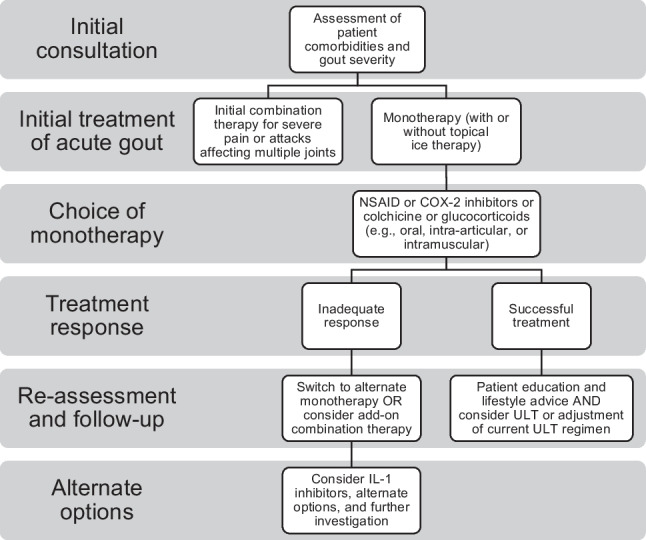
Consensus recommendations on the management of acute gout attack [6]. *COX*, cyclooxygenase; *IL*, interleukin; *NSAID*, non-steroidal anti-inflammatory drug; *ULT*, urate-lowering therapy

First-line options include colchicine, non-steroidal anti-inflammatory drugs (NSAIDs), or glucocorticoids (e.g., oral, intra-articular, or intramuscular). The choice of treatment is often dependent on the patient’s comorbidities and overall disease severity [8, 10, 13–20]. Initial combination therapy may be needed for patients experiencing severe pain or attacks affecting multiple joints (Fig. 1) [21], although the combination of an NSAID with systemic glucocorticoid is not recommended because of their additive toxicity.

#### Statement 1

“Acute flares of gout should be treated as early as possible, preferably within 12 h. Patients should be educated to self-medicate at the first warning symptoms.”

Level of evidence: 1B

Early management of flares (i.e., by self-initiated medication) is widely recommended by treatment guidelines based on high-grade evidence [8, 10, 14–17]. Anti-inflammatory therapy offers relief from pain and resolution of joint inflammation when administered within 12 h of an attack. In particular, low-dose colchicine self-administered within 12 h of flare onset has been shown to significantly reduce baseline pain without the associated gastrointestinal side effects associated with high-dose regimens [22]. Prompt treatment might also ameliorate the risk for cardiovascular events recently found to be associated with gout flares [23].

#### Statement 2

“Colchicine, NSAIDs, or glucocorticoids (oral, intra-articular, or intramuscular) are recommended as first-line therapy for gout flares.”

Level of evidence: 1B

Colchicine can be given at a loading dose of 1 mg, followed by 0.5 mg 1 h later on day 1; subsequent doses can be given at 0.5 mg twice or three-times daily until symptoms subside, or intolerable side effects occur. A gout flare generally warrants a higher dose of NSAID. However, treatment should be continued for the shortest possible duration needed to fully control the attack (typically 3–5 days) and to prevent recurrence. One potential advantage of NSAIDs is their analgesic effect, which may reduce pain before inflammation subsides. The typical glucocorticoid regimen is oral prednisolone 30 mg/day or 0.5 mg/kg for 5 days or tapered over 7–14 days. Joint aspiration and injection of intra-articular glucocorticoids are particularly indicated in flares affecting large joints or where there are other differential diagnoses [8, 10, 14–20, 24, 25].

#### Statement 3

“The choice of drug(s) should be based on the presence of comorbidities, patient's previous response to treatments, the severity of flares, and the number and type of joint(s) involved.”

Level of evidence: 1B

Colchicine dose should be reduced in patients with renal impairment and avoided in patients with severe renal impairment (glomerular filtration rate [GFR] < 30 mL/min) or in those receiving strong P-glycoprotein and/or cytochrome P450 (CYP) 3A4 inhibitors such as cyclosporin or clarithromycin. The concomitant use of statins may also increase the risk of myopathy and rhabdomyolysis. Evidence does not support the preferred use of any one NSAID, including cyclooxygenase-2 inhibitors, over another, so selection should be based on the patient’s prior response and concern over specific side effects. NSAIDs should be avoided in patients with renal impairment. Finally, systemic glucocorticoids are useful in acute, severe, and/or polyarticular attacks. A single dose of intramuscular glucocorticoids can be given, especially when a more rapid effect is desired. Intra-articular glucocorticoid injections are useful in patients with severe attacks involving one or two joints, especially in large weight-bearing joints [8, 10, 14–20, 24, 25].

#### Statement 4

“Use topical ice as an adjuvant therapy for pain relief.”

Level of evidence: 1B

In a small, randomized study of 19 patients with acute gout, the group treated with ice reported greater reduction in pain compared with patients in the control group. Both groups were also given prednisolone and colchicine [26].

#### Statement 5

“Interleukin-1 (IL-1) inhibitors can be considered for treatment of gout flare in patients who have inadequate response to or are contraindicated for standard treatment (including colchicine, NSAIDs, and/or glucocorticoids). IL-1 inhibitors are contraindicated in patients with active infection.”

Level of evidence: 1B

IL-1 inhibitors may be used in refractory polyarticular or tophaceous gout or for patients who are unable to tolerate conventional therapy for acute flares. These agents have been evaluated in case series and small randomized controlled trials/based on a low to moderate level of evidence. These agents may be an option in patients with chronic kidney disease (CKD), but its associated potential infectious complications and cost/benefit ratio must be carefully considered. Anakinra, an IL-1 receptor antagonist that inhibits the activity of both IL-1α and IL-1β, is effective in reducing acute gout pain and inflammation and may be a reasonable option in patients with CKD [27–32]. Rilonacept, a soluble decoy receptor that binds to IL-1β, may be used to reduce the risk of recurrent attacks [33]. Canakinumab may be an effective option in reducing both pain in acute attacks and the risk of recurrent attacks [34–36].

### Gout prophylaxis

Moderate quality evidence supports the administration of agents for flare prophylaxis, mainly with colchicine, among patients being treated with ULT during the first 6 months [8, 37–41].

#### Statement 6

“Prophylaxis with colchicine 0.5 mg once or twice daily for 3–6 months is recommended during initiation or up-titration of ULT.”

Level of evidence: 2B

Colchicine should be used with caution in patients with renal or hepatic impairment. In patients receiving medications that inhibit CYP3A4 and/or the P-glycoprotein efflux pump, dosage adjustment or avoidance of colchicine is warranted [42].

#### Statement 7

“In patients who cannot tolerate colchicine, a low-dose NSAID can be considered as an alternative for prophylaxis after weighing the risks and benefits.”

Level of evidence: 2B

Alternate regimens, such as low-dose NSAIDs, may be of benefit in patients who may present with relative or absolute contraindications to colchicine therapy. However, the routine use of low-dose NSAIDs, glucocorticoids, as well as IL-1 inhibitors for gout prophylaxis is not currently supported by strong evidence [8, 43–46]; therefore, careful consideration of the risks and benefits (i.e., not only evaluation of contraindications) may be clinically relevant in certain populations.

### Indications for urate-lowering therapy

Clinical trials have demonstrated that ULT is effective in treating patients with recurrent flares, tophi, urate arthropathy, and/or renal stones [47–69].

#### Statement 8

“ULT should be discussed with all patients diagnosed with gout.”

Level of evidence: 5

As suboptimal understanding of gout and its treatment poses as a common barrier to gout care, the core group considered early introduction of ULT to patients would serve as an important education strategy in the management of gout.

#### Statement 9

“ULT should not be routinely recommended on the first attack of gout in patients without comorbidities.”

Level of evidence: 5

The usefulness of ULT in patients diagnosed with gout is most apparent in patients with recurrent gouty arthritis and with associated signs and symptoms of either tophaceous or non-tophaceous arthropathy [69, 70]. It is not particularly advised for patients who experience a single, isolated episode of pain or joint swelling to receive ULT. A study by Yu and Gutman documented that only 62% of patients with gout had a second attack within a year [71]. A Canadian study showed that gout treatment only becomes cost-effective in patients who experience at least three attacks per year [70]. Therefore, ULT should be reserved for patients who have documented organ affectation or in those who experience frequent flares. On the other hand, there is also fear of delaying the initiation of ULT until the second or third attack, exposing patients to a higher crystal load, which may be deleterious for the cardiovascular system and kidneys [8, 10]. Therefore, the panel considers that in selected patients who prefer to start treatment after thorough discussions on the risks and benefits, ULT might remain an option.

#### Statement 10

“ULT should be recommended to all gout patients with tophi, radiographic damage related to gout, OR recurrent attacks (≥ 2 times per year).”

Level of evidence: 1B

High-quality evidence from a randomized controlled trial and as confirmed by a meta-analysis demonstrated that ULT can reduce tophi size and numbers [48, 54]. Various authorities set a range of 0–3 attacks a year, but most guidelines have adopted a strategy of initiating ULT after two attacks per year [8, 10, 14–17].

#### Statement 11

“ULT can be considered in patients with gout after the first attack with urolithiasis, more than one flare but with infrequent attacks per year, or in those with renal impairment.”

Level of evidence: 1B

In patients with a history of urolithiasis or those experiencing calcium oxalate renal stones and/or hyperuricosuria, ULT has been documented to provide benefit by lowering 24-h urinary uric acid excretion more significantly than placebo [55, 56]. The 3-year incidence of stone-related events was shown to decrease in those treated with allopurinol vs. those treated with placebo [57]. However, the benefit of ULT appears to decrease in patients with less frequent flares compared with when ULT is prescribed to patients with more frequent flares. Dalbeth et al. demonstrated that patients with at the most two previous flares and no more than one attack in the preceding year were less likely to experience a subsequent flare (30% with febuxostat vs. 41% with placebo; *p* < 0.05) [58]. Hence, the number of gouty attacks should be carefully considered before the decision to start ULT is reached. In addition, renal disease should likewise influence the decision to start ULT. Both allopurinol and febuxostat has been documented to lower 24-h urinary uric acid excretion [56]. Allopurinol is also known to slow the progression of CKD in hyperuricemia patients. Because there is a higher likelihood of gout progression and tophi development in patients with CKD compared with those with no renal impairment, this population may benefit from early initiation of ULT [59–64].

#### Statement 12

“ULT may be considered in patients with gout after the first attack with very high serum urate level (> 0.54 mmol/L [9 mg/dL]) or young onset age (age < 40 years).”

Level of evidence: 1B

The risk of gout rises sharply when serum urate levels are above 0.5 mmol/L (8.4 mg/dL). Patients with markedly elevated serum urate levels > 0.54 mmol/L (9 mg/dL) are more likely to experience gout progression than those with lower levels [66, 67, 69, 72]. Young age is a marker of severity and may be associated with an inborn error of metabolism or dysfunctional variant in the urate transporter [73–76].

### Treatment target of urate-lowering therapy

There is sparse evidence from randomized trials that establishes the treat-to-target approach in gout. However, data from observational studies, including longer extension studies, appear to suggest that SUA < 0.36 mmol/L (6 mg/dL) was associated with reduced gout flares [69, 77–81].

#### Statement 13

“For patients on ULT, serum urate level should be monitored and maintained below 0.36 mmol/L (6 mg/dL). A target SUA of <0.30 mmol/L (5 mg/dL) is recommended for patients with tophaceous gout.”

Level of evidence: 3

The aim of ULT is to reduce and maintain the serum urate level to prevent further urate crystal formation, to dissolve existing crystals, and to prevent recurrent flares. The lower the serum urate level, the greater the velocity of crystal elimination [69, 77–82].

#### Statement 14

“When the clinical tophi have resolved, the serum urate level may be maintained between 0.30 mmol/L (5 mg/dL) and 0.36 mmol/L (6 mg/dL).”

Level of evidence: 3

The recommendation of a less stringent targeted serum urate level of between 0.30 mmol/L (5 mg/dL) and 0.36 mmol/L (6 mg/dL) after clinical tophi have resolved is based on the possibility of adverse effects that may be associated with a very low SUA level [69, 77–82]. Studies have shown that hyperuricemia might protect against various neurodegenerative disorders such as Parkinson’s disease and dementia, among others, and should therefore be a strong consideration among elderly patients with gout [83, 84].

### Precautions in prescribing allopurinol

The human leukocyte antigen (HLA)-B*5801 haplotype is the strongest risk factor for allopurinol-induced severe cutaneous adverse reactions (SCARs). Allopurinol-induced SCARs include drug hypersensitivity syndrome, Stevens–Johnson syndrome (SJS), and toxic epidermal necrolysis. Patients who experience SCARs after ULT often have a poor prognosis. However, there are insufficient data to establish firm recommendations for cost-effective screening in populations with low allele frequency [17, 85–98].

#### Statement 15

“Allopurinol should be avoided in patients who have tested positive for HLA-B*5801 allele.”

Level of evidence: 2B

As mentioned, HLA-B*5801 haplotype is the strongest risk factor for allopurinol-induced SCARs. Screening for HLA-B*5801 is beneficial in populations with high rates of allopurinol-induced toxic epidermal necrolysis/SJS, especially in populations with a higher frequency of the allele (≥ 5%) [92].

#### Statement 16

“Screening for the HLA-B*5801 allele should be considered for some patients of Asian descent (e.g., Han Chinese, Korean, Thai) and for African American patients, and patients with risk factors to develop allopurinol-induced SCAR, before starting of allopurinol. Risk factors included patients who are ≥60 years old or with renal insufficiency (CKD stage ≥3).”

Level of evidence: 2B

Certain populations particularly benefit from genetic testing for HLA-B*5801 before allopurinol administration because of the high potential cost-effectiveness of this intervention. In a local study, Wong et al. suggested that pre- treatment HLA-B*5801 screening is cost-effective in Chinese patients with CKD to prevent allopurinol-induced SCARs [99]. Apart from consideration of ethical, legal, and social implications to land at informed policy decision-making, awareness of risk factors, such as age or renal insufficiency, may be necessary to justify the appropriateness of the utilization of this test [92–94, 96]. Stamp et al. posit that a starting dose of 1.5 mg per unit of estimated GFR may reduce this risk [98]. The group suggests the use of alternative ULTs (e.g., febuxostat) in these patients.

### Use of urate-lowering therapy

The use of ULT for the prevention of recurrent gout flares and disease progression and the treatment of tophi is supported by clinical trials/high level of evidence. The choice between xanthine oxidase inhibitors (XOIs) and/or uricosurics is dependent on a patient’s clinical picture. These recommendations are based on a moderate to high level of evidence [100–115].

#### Statement 17

“A xanthine oxidase inhibitor (allopurinol or febuxostat) is the preferred agent for all patients with gout.”

Level of evidence: 1A

Allopurinol should be considered as the first-line agent especially in patients with pre-existing major cardiovascular diseases. The starting and maximum dosage of allopurinol should be adjusted according to creatinine clearance in patients with renal impairment [100–115]. However, in individuals who are observed to tolerate allopurinol, there is evidence that gradually increasing the dose above doses based on kidney function is safe and effective in those with chronic kidney disease [116, 117]. There is no evidence that limiting the maintenance dose reduces the risk of allopurinol hypersensitivity syndrome [118].

#### Statement 18

“If the targeted serum urate level cannot be achieved by allopurinol, febuxostat should be considered. Alternatively, combination therapy with a uricosuric agent can be considered in patients without severe renal impairment (GFR <30 mL/min).”

Level of evidence: 1A

Observational and cohort studies have shown that the use of febuxostat in patients with CKD stages 3–5 was not associated with increased adverse events but conferred more significant reduction in serum urate levels vs. allopurinol [100, 103]. Results of the Cardiovascular Safety of Febuxostat and Allopurinol in Participants With Gout and Cardiovascular Comorbidities (CARES) trial, highlight the noninferiority of febuxostat vs. allopurinol as regards rates of adverse cardiovascular events [119]. Although this study posits that all-cause mortality and cardiovascular mortality were higher with febuxostat than with allopurinol, the long-term Febuxostat versus Allopurinol Streamlined Trial (FAST) study established that after a median follow-up of about 4 years, febuxostat was not associated with a higher risk of death or serious adverse events compared with allopurinol [120]. Hence, the presence of pre-existing major cardiovascular diseases should not preclude the use of febuxostat in patients who fail to achieve the treatment target with allopurinol [101–103, 121].

The use of uricosuric monotherapy is not preferred unless patients present with a contraindication or intolerance to both XOIs. The use of allopurinol or febuxostat with a uricosuric agent decreases the urate concentration in urine and thus the risk of urolithiasis [109–115]*.*

### Lifestyle modification

Dietary interventions limiting red meat, seafood, sugary beverages, and alcohol have been the cornerstone of lifestyle management among patients who have gout. Also, moderate to heavy physical activity and overall fitness have been found to be associated with a lower incidence of gout and hyperuricemia [122–125].

#### Statement 19

“Patients should be advised to avoid alcohol, fructose, or sugar-sweetened beverages, and decrease dietary protein source from meat and seafood.”

Level of evidence: 2B

A diet that restricts alcohol and purine-rich and fructose-rich foods, while maintaining the intake of low-fat dairy products, healthy protein sources, and vegetables, has been observed to lower the risk of incident gout [122]. Ethanol has been found to facilitate increased urate production through acetate metabolism and enhanced adenosine triphosphate turnover [126–128]. Moreover, beer contains purines that have been found to intensify the plasma concentration of uric acid [129, 130]. Substantial intake of red meat and seafood, or food stuffs with high purine content, has been likewise positively correlated with the development of hyperuricemia and gout flares [131]. In a study of vigorously active men, those with higher alcohol intake (per 10 g/day) had a higher relative risk (RR) of 1.19 (95% confidence interval [CI]: 1.12–1.26; *p* < 0.0001) and those with a higher meat consumption (per servings/day) had a higher RR (1.45; 95% CI: 1.06–1.92; *p* = 0.002), whereas those with greater fruit intake (per piece/day) had a lower RR (0.73; 95% CI: 0.62–0.84; *p* < 0.0001). Men who consumed more than 15 g of alcohol (in particular beer) per day had a 93% higher risk and higher rates of SUA levels than those who had abstained, and men who averaged more than two pieces of fruit/day had cut their risk in half compared with those who ate < 0.5 pieces of fruit/day and had comparative body mass index (BMI) levels (RR: 1.19; 95% CI: 1.15–1.23; *p* < 0.0001) [123, 132, 133]. Fructose metabolism leads to an increase in purines, which are converted into uric acid. Also, long-term fructose administration suppresses urinary excretion of uric acid leading to hyperuricemia [134]. The consumption of five to six servings of sugar-sweetened soft drinks was associated with an increased multivariate RR of 1.29 (95% CI: 1.00–1.68) compared with one serving a month [135].

#### Statement 20

“Weight reduction is recommended if BMI > 25 kg/m^2^.”

Level of evidence: 2B

A prospective cohort study of over 40,000 men with excess adiposity and other key modifiable factors suggests that weight control has the potential to prevent most incident gout cases among men. The authors of the study state that men with obesity may not respond to gout therapy unless weight loss is achieved [122]. In a separate study, the risk of gout was noted to be 16-fold greater for men with BMI > 27.5 kg/m^2^ than in those who had a BMI < 20 kg/m^2^. In addition, compared with those who were least active or fit, men who ran ≥ 8 km/day or > 4.0 m/s had 50% and 65% lower risk of gout, respectively [136]. A systematic review by Nielsen et al. showed that low to moderate grade evidence supports weight loss in overweight gout patients achieve SUA targets and have fewer gout attacks at medium-term or long-term follow-up on SUA [137]. Overall, obesity has an impact on both incidence and control of gout.

## Discussion

This set of HKSR consensus recommendations for gout management encompasses lifestyle and medical interventions that offer guiding provisions for primary care and specialist clinicians to aid in lowering the risk of gout and hyperuricemia in their patients. The 60 members of the voting faculty represented practicing rheumatologists, renal medicine specialists, general practitioners, and internists in the locality. To date, this paper is the first consensus document on gout management involving most practicing rheumatologists in Hong Kong. The voting panel included over 80% of local practicing rheumatologists. The final recommendation statements consider existing evidence and local viewpoints to aid in the optimization of gout and hyperuricemia control in Hong Kong. The individual recommendations have been enumerated in logical sequence and are intended to guide practitioners in administering appropriate medical treatment coupled with non-pharmacologic interventions, involving several aspects of care that are of particular concern in the locality. For instance, screening for HLA*B-5801 was emphasized and may be justified as standard practice in our clinical setting. In addition, the introduction of newer agents in our locality, such as IL-1 inhibitors for acute gouty attacks in complicated cases and the use of ULT for chronic cases, was included. In most cases, gout and hyperuricemia can be managed in the primary care setting. Patients who are refractory to standard care may require specialist management and should be referred as and when necessary.

Treatment of acute gout remains largely dependent on the severity and the clinical profile of patients. Prompt therapy of acute attacks with colchicine, NSAIDs, or glucocorticoids is considered, but with careful watch over comorbidities, especially renal disease. Although evidence supports the use of IL-1 inhibitors for gout prophylaxis [43–46], the use of canakinumab and rilonacept may be precluded by their costs and putative infection risks. The group has considered these factors and emphasizes that the availability of these agents should be taken into consideration when administered as first-line prophylaxis for gout. Prophylaxis is currently limited to colchicine and low-dose NSAIDs given to patients during initiation or up-titration of ULT. ULT is effective in treating patients with recurrent flares, tophi, urate arthropathy, and/or renal stones, but not in all instances. A review of the patient’s age, risk factors, current treatments, organ affectations, signs and symptoms, and required treatment targets should be conducted at the onset. Allopurinol remains a popular choice for ULT across various populations. HLA-B*5801 screening should be considered in high-risk patients before initiation of allopurinol. The cost-effectiveness of HLA-B*5801 screening for preventive management of SCARs should be evaluated before the determination of the necessity for this intervention in low-risk patients across the different centers and hospitals in Hong Kong. Febuxostat may be considered as second-choice therapy in those with known allopurinol-induced hypersensitivity or adverse reactions. Recent guidelines recommend switching from febuxostat to other ULT options in patients with a history of cardiovascular disease or new cardiovascular events based on the results of the CARES trial [10, 119]. However, the results of this study pose uncertainties, and data from subsequent exploratory analyses of the CARES trial countered the contended association between febuxostat use and an increased mortality risk [138, 139]. More recent studies have also established the safety of febuxostat after 4 years of use [120, 121]. Hence, the core members of the steering committee suggest that the presence of pre-existing major cardiovascular diseases should not preclude the use of febuxostat in indicated patients.

The panel did not achieve consensus on recommendations pertaining to co-administration of non-gout medications with ULT after two rounds of voting. This may be related to the relative lack of strong evidence [140–147]. However, as reported by an epidemiologic study, the RRs of associated incident gout were lower with current use of calcium channel blockers and losartan. Hence, in gout patients, calcium channel blockers and losartan are preferred over other antihypertensive drugs, e.g., loop or thiazide diuretics, beta-blockers, angiotensin-converting enzyme inhibitors, and non-losartan angiotensin II receptor blockers [140]. For patients with gout who are receiving low-dose aspirin (< 300 mg daily) alone or in combination with clopidogrel or ticagrelor for prevention or treatment of cardiovascular disease, it is advisable to monitor serum uric acid levels regularly, and make appropriate ULT adjustment accordingly [141, 142]. The long-term efficacy and safety of slow oral desensitization to allopurinol are established for gout patients with maculopapular eruptions who cannot be treated with uricosurics or another ULT; however, it is not recommended in patients with severe hypersensitivity to allopurinol. The panel recognizes that desensitization protocols are not commonly used, with most currently practicing rheumatologists having limited experience in these protocols [97].

Finally, lifestyle modification is a cornerstone in the management of hyperuricemia and gout, and relevant patient education regarding diet and exercise should be espoused when treating these patients.

Local registry studies and trials looking at various aspects of gout therapy may be worthwhile to consider. Other areas of gout treatment, such as the usefulness of surgery and other clinical aspects of care, should be investigated in future studies. These new data may be useful in reshaping upcoming recommendations to support the improvement of gout care in Hong Kong. Likewise, optimization of treatment approaches and holistic management of other metabolic conditions may help in the overall improvement of patients and should become the focus of future guidelines.

## Data Availability

The data that support the findings of this study are available with from the corresponding author with permission of the Hong Kong Society of Rheumatology.
